# In Vitro Propagation, Phytochemical and Neuropharmacological Profiles of *Bacopa monnieri* (L.) Wettst.: A Review

**DOI:** 10.3390/plants9040411

**Published:** 2020-03-26

**Authors:** Partha Sarathi Saha, Sayantika Sarkar, Rajendran Jeyasri, Pandiyan Muthuramalingam, Manikandan Ramesh, Sumita Jha

**Affiliations:** 1 Center of Advanced Study, Department of Botany, University of Calcutta, 35, Ballygunge Circular Road, Kolkata 700019, India; parthasarathisaha11@gmail.com (P.S.S.); sarkar.sayantika89@gmail.com (S.S.); 2 Department of Biotechnology, Science Campus, Alagappa University, Karaikudi 630 003, India; jeyasri8220@gmail.com (R.J.); pandianmuthuramalingam@gmail.com (P.M.); mrbiotech.alu@gmail.com (M.R.)

**Keywords:** *Brahmi*, saponins, micropropagation, pharmacological activity, bioreactor

## Abstract

*Bacopa monnieri* has been used as a reputed drug in the Indian traditional ayurvedic system for centuries. This medicinal herb with important phytopharmaceuticals has been popularly known as “Brahmi”. In recent years, *B. monnieri* has been extensively studied for its bioactive constituents, constituents responsible for memory enhancing effect, and also its diverse other useful effects. It possesses many pharmacological activities such as antioxidant, gastrointestinal, endocrine, antimicrobial, anti-inflammatory etc. The plant has been also used for the treatment of neurological and neuropsychiatric diseases. Due to its multipurpose therapeutic potential, micropropagation using axillary meristems and de novo organogenesis has been extensively studied in the species and is being reviewed. High frequency direct shoot organogenesis can be induced in excised leaf and internode explants in the absence of exogenous phytohormones and the rate of induction is enhanced in the presence of exogenous cytokinins, supplements, growth regulators, etc. Using explants from tissue culture raised plants, direct shoot regeneration leading to production of more than 100 rooted plants/explant within 8–12 weeks period with 85%–100% survival in the field after acclimatization can be expected following optimized protocols. Bioreactor based micropropagation was found to increase the multiplication rate of shoot cultures for the commercial propagation of *B. monnieri* plants. The maximum content of bacosides has been recorded in shoot biomass using an airlift bioreactor system. Further studies for the biosynthesis of bacosides and other secondary metabolites need to be conducted in the species utilizing untransformed shoot cultures in bioreactors.

## 1. Introduction

*Bacopa monnieri* L. (Family: Scrophulariaceae), commonly known as ‘Brahmi’, is a perennial and semi- succulent herb which grows in wet, damp, and marshy areas throughout India. It is an ancient medicinal plant with a legendary reputation as a vitalizer of the memory. For 5000 years, it has been used in India to treat epilepsy and insomnia and to reduce herbal sedation and anxiety [1]. This plant is recommended by Indian Materia Medica (Bhavaprakasha Nighantu AD 1500) for the treatment of a wide range of mental conditions, including anxiety, poor cognition, lack of concentration, insomnia, insanity, depression, psychosis, epilepsy, and Alzheimer’s disease [2,3,4,5]. Commercially available *B. monnieri* preparations increase memory concentration and development and enhance brain function in both young and older people. The plant was also used as a cardiac tonic, digestive aid in India and Pakistan, and has been found to improve respiratory function in cases of bronchoconstriction. Clinical studies affirm that bacopa based formulations have positive effects on the reconstruction of mental functions in children suffering from attention deficit hyperactivity disorder (ADHD), and contribute to the enhancement of cognitive functions in stroke and epilepsy patients [6,7,8]. Bacosides and triterpenoids which belong to the saponins are compounds attributed to the above activities [9].

In recent studies, *B. monnieri* has been placed second in a priority list of the major Indian medicinal plants assessed on the basis of medicinal significance, potential candidate, and commercial value for further research and development [10,11]. It possesses numerous pharmacological activities, including anxiolytic [12], anti-neoplastic [13], anti-depressant [14], anti-ulcerogenic [15], adaptogenic [16], anti-convulsant [17,18,19] etc. This herb acted as a mental chelating agent in the bloodstream which can eliminate any excess of toxic metals. It is also used for the removal of heavy metals such as chromium and cadmium in phytoremediation. Since *B. monnieri* is the lone herbal source of bacosides, it is harvested at a very high rate from its natural habitat by pharmacologists and herbal traders. Due to its immense diverse medicinal importance, generation of mass propagation of the plants as well as other alternative strategies for biotechnological production of its active principles, the bacopa saponins, have attracted the attention of researchers resulting in large number of publications on in vitro propagation in the species. The morphogenic potential of explants of *B. monnieri* favors its use as a model plant for in vitro studies on the expression of transgenes on organogenesis in vitro and functional studies on bacoside synthesis in vitro, avoiding the effects of exogenous phytohormones.

## 2. Axillary Shoot Proliferation

The multiplication of shoots for the clonal propagation of *B. monnieri* have been reported using pre-existing meristems such as nodes and shoot tips derived from both ex vitro (~45% reports) and in vitro grown plants (~16% reports). Among the two types of explants used, nodes have been found to be the better choice of explants for in vitro shoot multiplication (Table 1). The rate of propagation was found to depend on the type and concentration of cytokinins used in the culture medium. Although three different types of cytokinins such as BA, (N6-benzyladenine) Kn (Kinetin), and TDZ (Thidiazuron), have been used, maximum reports on in vitro propagation (~57%) involved the use of BA alone or in combination with other hormones, whereas the effect of Kn alone or in combination with other PGR was investigated in only 9% of published reports. Apart from hormones, other additives such as algal extract, organic supplements etc. have been used in ~12% of published reports to improve the rate of shoot induction in *B. monnieri*.

Tiwari et al. [43] obtained ~20 shoots/nodal explant in MS medium supplemented with 2 mg L^−1^ BA after four weeks of culture. When the medium was supplemented with lower levels of BA in combination with IAA, optimum shoot growth with an average shoot length of 6.8 ± 0.6 cm was observed after 28 days of culture. The rate of shoot multiplication from nodal explants was further improved (~42 shoots/explant) when 6.8 µM TDZ was used in the medium [21]. Sharma et al. [36] obtained 41shoots/nodal explant through axillary bud break at very low concentration of BA (0.1 mg L^−1^). Tiwari et al. [43] showed a significant improvement in axillary shoot induction (50 axillary shoots/explant) from nodal explants in response to the combined actions of 200 mg dm^−3^ TMP and 200 mg dm^−3^ BVN in MS medium. Additionally, the induction of adventitious shoot buds (30) was also reported from these nodal explants although no histological evidences were provided [43]. Therefore, it is unclear whether these adventitious shoot buds were induced de novo or were formed as a result of the multiplication of pre-existing meristems. Wangdi and Sarethy [62] used liquid MS medium supplemented with Kn for shoot multiplication and obtained 36–75 rooted shoots along with adventitious shoots/nodal explant after 7 days of culture. Recently, Behera et al. [54] obtained 100% shoot bud induction in nodal explants on MS medium supplemented with 3.0 mg L^−1^ BA, eventually improving the rate of multiplication using 1.0 mg L^−1^ GA_3_ to 114.2 shoots/explant with shoot length 6.4 cm after three weeks.

The use of algal extract as an alternative of PGR regulator for in vitro propagation of *B. monnieri* was demonstrated by Banerjee and Modi [32] and Pothiaraj et al. [61]. The effect of PGRs and seaweed liquid extracts (SLEs) on in vitro shoot induction using encapsulated shoot tips of *B. monnieri* was elegantly demonstrated by Rency et al. [64]. They obtained 92 shoots/explant (with 7.9 cm of mean shoot length) after one week of culture on MS medium containing 60% of *G. salicornia* extract. After two weeks of culture on the same medium, enhanced multiplications (with ~145 shoots) were obtained.

Root induction and development in microshoots of *B. monnieri* has been successful in most micropropagation methods using full- or half-strength MS medium with or without an auxin (IBA or NAA at 0.5 or 1 mg/L). Tiwari et al. [24] reported that the root number and length was maximum in 4.9 μM IBA supplemented medium. Hardening and subsequent field establishment has been achieved in a majority of reports on micropropagation in the species (Table 1). Recently, biochemical and histological changes during the adventitious in vitro rooting of *B. monnieri* micro shoots revealed a significant role of enzymes, sugars, and phenols during different phases of rooting [68].

## 3. Organogenesis

**Figure 1:**
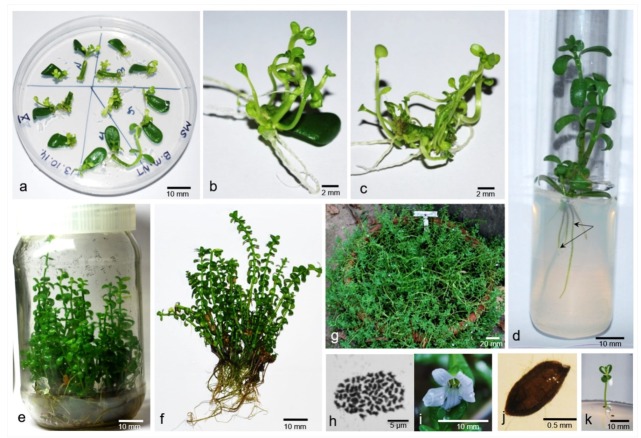
Micropropagation of *B. monnieri* in MS basal medium. (**a**) adventitious shoot induction in leaf and internode explant after 3 weeks of culture, (**b**) and (**c**) shoot proliferation after 6 weeks of culture, (**d**) rooted micro shoots, (**e**) rooted micropropagated plants from single leaf explant, (**f**) rooted plantlets prior to transfer to soil, (**g**) Acclimatized plants with flowers after 6 months of field transfer, (**h**) somatic metaphase plate in root cell showing 2n = 64 chromosomes. (**i**,**j**) flower, seed respectively and (**k**) in vitro germination of seed obtained from *ex vitro* micropropagated plants.

In *B. monnieri* various researchers have reported adventitious bud formation and shoot organogenesis in vitro. However, there are very few reports on callus induction and indirect shoot organogenesis in the species [37,47,53]. The morphogenic potential of plant cells leading to whole plant regeneration is known to depend on both endogenous and exogenous factors [69]. However, the expression of totipotency of cells of excised leaf and stem explants from in vitro *B. monnieri* plants in plant growth regulator (PGR) free medium makes this species a model system to study the causes of morphogenesis *in vitro* Figure 1 [65] The rate of shoot organogenesis in *B. monnieri* among other factors also depends on type and position of explants [65] while Joshi et al. [34] reported both direct and indirect shoot organogenesis depended on the orientation of explants on the shoot induction medium.

Researchers have used explants derived from *ex vitro* and in vitro grown plants for micropropagation of *B. monnieri* (Table 1). The morphogenic potential of leaf and internode explants was found to enhance significantly in terms of both the frequency and rate of shoot organogenesis when cultured in the presence of exogenous hormones or growth regulators.

### 3.1. Organogenesis from In Vitro Derived Explants

Nearly 20% of the published reports on micropropagation of *B. monnieri* have used leaf and internode explants from in vitro grown plants for whole plant regeneration through direct shoot organogenesis. Tiwari et al. [21] reported direct adventitious shoot regeneration (~80 shoots/leaf explants) on MS [67] medium supplemented with BA (6-benzylamino purine). In a later publication, Tiwari et al. [24] reported the rate of adventitious shoot bud formation was enhanced (93 shoot buds/leaf explant in seven days) using TDZ instead of BA in shoot induction medium (SIM). After three subcultures on shoot multiplication medium (SMM) containing BA yielded a higher number (~130) of adventitious shoot buds/leaf explant.

The cytokinin- like effect of TDZ in vitro has been observed in the morphogenesis and regeneration in a large number of plants [70]. However, its mode of action and role in the inductive phase of morphogenesis in vitro is not known [71].

The promotive effect of TMP (Trimethoprim, an antibiotic) and BVN (Bavistin, a fungicide) on de novo shoot organogenesis was also reported [24]. Ceasar et al. [33] using TDZ and NAA (α-naphthaleneacetic acid) initially reported 49 and 56 shoots/internode and leaf explants could be induced, and the rate of shoot multiplication enhanced significantly (approximately 135 shoots/leaf explant and 112 shoots/internode explant) on transfer to SMM containing low levels of BA.

The promotive role of spermidine in combination with BA in adventitious shoot bud induction from mature leaves excised from in vitro propagated plants was reported couple of years back by [63]. Thus, using explants from tissue culture raised plants direct shoots regeneration leading to production of more than 100 rooted plants/explant within 8–12 weeks period with 85%–100% survival in the field after acclimatization can be expected following optimized protocols summarized in Table 1.

### 3.2. Organogenesis from Ex Vitro Derived Explants

Shoot regeneration using leaf and internode explants excised from ex vitro plants has been demonstrated in nearly 36% of the published reports. Optimum shoot regeneration (110 shoots/leaf explant) was reported by Binita et al. [26] within three weeks of culture on medium supplemented with BA and IAA (Indole-3-acetic acid). Using Kinetin (Kn) instead of BA, Praveen et al. [31] obtained shoot induction and proliferation to a maximum frequency of 155 shoots/leaf explant after 18 weeks. Kn has been used similarly to induce high frequency shoot regeneration in leaf explants by Umesh et al. [53], while shoot induction frequency was low with 2ip (N6-2-isopentenyl adenine) [72]. On the other hand, in internode explant, significantly higher shoot induction (324 shoots/internode explant) was obtained in a combination of BA and Kn after three subcultures [29]. It can be concluded that both internode and leaf explants derived from *ex vitro* plants show significantly higher potential for micropropagation (155–324 shoots/explant) than similar explants derived from in vitro plants.

## 4. Somatic Embryogenesis

There are only a few reports on somatic embryogenesis reported in *B. monnieri*. Tiwari et al. [21] reported plant regeneration through embryogenic callus mediated somatic embryogenesis followed by the development and maturation of somatic embryos (SEs) on MS medium comprising 0.5 mg L^−1^ BA. Khilwani et al. [60] described both types of morphogenesis, i.e., shoot organogenesis as well as somatic embryogenesis from in vitro derived leaf explants cultured on MS medium supplemented with 12.5 μM BA and 1.0 μM 2,4-D. The addition of 250 mM sucrose in this medium was found to enhance somatic embryogenesis (77%) probably due to high osmotic stress and/or the source of energy required for somatic embryogenesis. Although histological evidence of different stages of somatic embryo differentiation has been provided [21,60], the evidence of SE developmental stages is lacking and needs to be studied in detail in *B. monnieri*. Callus-mediated somatic embryogenesis in leaf explants obtained from 2-months old greenhouse plants was reported by Sharath et al. [28]. The optimum frequency of organized embryoids/callus clump (96%) and differentiation of embryoids (42/callus clump) occurred in medium supplemented with 0.5 mg L^−1^ 2,4-D. Germination, maturation, and further multiplication of these embryoids occurred on MS basal medium with or without BA.

## 5. Clonal Fidelity and Phytochemical Analysis in In Vitro Propagated Plants

Somaclonal variations are known to occur in tissue culture raised plants particularly where whole plants regenerated via callus induction and dedifferentiation [73] using different explants [74]. Cytogenetic analysis and molecular markers are frequently used to determine the genetic fidelity of in vitro propagated plants in many species. Cytogenetic analysis of micropropagated plants of *B. monnieri* (2n = 64) have not been reported to date owing to perhaps small chromosome size and high number [75]. However, it is possible to avoid somaclonal variations often associated with tissue culture regeneration in vitro in *B. monnieri* as large scale propagation via direct organogenesis as well as axillary bud multiplications has been optimized by a large number of researchers.

However, there are few reports where clonal fidelity of in vitro propagated plants of *B. monnieri* has been demonstrated [33,60,64,76]. Recently, a novel technique for the clonal propagation of true-to-type plants in *B. monnieri* has been reported by Faisal et al. [66]. Using TDZ pulse treated nodal explants, high frequency regeneration with optimum multiplication (43 shoots per explant) were obtained after eight weeks of culture from explants treated with 20 uM TDZ. Microshoots that rooted in growth regulator free media were successfully acclimatized with 97% survival ex vitro. The genomic stability of the micropropagated plants were confirmed by flow cytometry and single primer amplification reaction (SPAR) for the first time. They reported no significant variation in nDNA content and ploidy level between micropropagated plants and control [66].

Analysis of phytochemicals among in vitro propagated plants of *B. monnieri* was performed in nearly 18% of the published reports, mostly general phytochemical analysis Table 1 [10,23,26,40]. Praveen et al. [31] reported that accumulation of bacoside A in shoots grown on Kn supplemented medium was 4.5-fold higher compared to shoots developed on medium not supplemented with growth regulators. Furthermore, they suggested that liquid cultures are preferable for the production of bacoside A. Parale et al. [35] reported the influence of organic supplements in the culture medium on production of bacoside A in both shoot and callus biomass. The inclusion of 100 µM pyruvic acid showed the maximum accumulation of bacoside A in shoot (4.0 times) as well as callus biomass (3.8 times) than control. Umesh et al. [53] detected bacopaside I and II in regenerated shoots and found that Kn favored maximum accumulation of bacopaside II whereas the amount of bacopaside I was highest in the shoots regenerated on MS medium supplemented with BA. Largia et al. [77] reported an increase in bacoside A production by the synergism of Methyl jasmonate (MJ) and salicylic acid (SA) in shoot cultures of *B. monnieri.*

## 6. Culture in Bioreactors

Bioreactor based micropropagation was found to increase the multiplication rate of shoot cultures using nodal segments, for commercial propagation of *B. monnieri* plants [38,56]. Jain et al. [38] reported enhanced biomass production in Growtek^®^ bioreactor with 10% aeration using nodal explants. Maximum growth index (10.0) with ~2000 shoots/L and 16.5 g/L DW was recorded when cultured on liquid MS medium fortified with 2.5 mg L^−1^ BA and 0.01 mg L^−1^ IAA. In another study, using airlift bioreactor, nearly 443 shoots proliferated from ~48.33 excised shoots after four weeks of culture in liquid MS medium (1.5 l) supplemented with 1 mg L^−1^ BA (Sharma et al. [56]. Investigations on biosynthesis of bacosides (A_2_ + A_3_) in in vitro shoot cultures of *B. monnieri* grown in two bioreactor systems, i.e., Growtek^®^ (1 L) and modified bench top air lift bioreactor (5 L) revealed that the maximum content was obtained in shoot biomass using ALB system (10.15 mg g^−1^ DW) as compared to Growtek^®^ culture (6.08 mg g^−1^ DW) due to higher aeration facility for improved oxygen transfer during bacoside synthesis [56]. Thus, further studies using bioreactors can be explored for the commercial production of bacosides in mass propagated shoot cultures.

## 7. Bioactive Compounds in *B. monnieri*

The pharmacologically active compounds of bacopa include saponins, steroids, and alkaloids. Comprehensive studies first confirmed that *B. monnieri* isolated the alkaloid ‘brahmine’ [78]. In the same year, other alkaloids such as herpestine and nicotine were also reported [79]. D-mannitol and a saponin, hersaponin, and potassium salts were subsequently isolated [80]. Bacoside A, known as 3-(a-L-arabinopyranosyl)-O-b-D-glucopyranoside-10, 20-dihydroxy- 16-keto-dammar- 24-ene, is the major chemical entity shown to be responsible for neuropharmacological effects and the nootropic activity or antiamnestic effect of *Bacopa* [81], Bacoside A typically co-occurs with Bacoside B; the latter only varies in optical rotation probably in an artefact produced during the bacoside A isolation process [82]. The major chemical components isolated and characterized by different major spectral, chemical and 2D NMR studies carried out by various research groups from the herbal alcoholic extract are dammarane type of triterpenoid saponins with jujubogenin andpseudojujubogenin as aglycones (Table 2). Bacosides yield a mixture of aglycones on an acid hydrolysis, bacogenin A1, A2, A3 [83]. Three new triterpenoid saponins of biological interest, bacopasaponins A, B, and C, pseudojujubogenin were isolated and a new dammarane type pseudojujubogenin glycoside, bacopasaponin D, was identified using chemical transformation and spectroscopic methods [12]. Two new pseudojujubogenin glycosides, known as bacopaside I and II, have been isolated from glycosidic fraction of the methanol [84]. Thereafter, three new saponins, called Bacopasides III, IV, and V, were isolated. Furthermore, three new phenylethnoid glycosides (monnierasides I to III) were identified from the glycosidic fraction of *B. monnieri* along with the known analogue plantainoside B [78].

## 8. Pharmacological Activities

*B. monnieri* is an excellent medicinal plant which offers numerous promising pharmacological activities that are useful in treating of many complex diseases/disorders. Some of the prominent bioactivities are anti-inflammatory, antiamneatic, nootropic, cardioprotective, neuroprotective/antioxidant, hepatoprotective, anti-Alzheimer’s, anti-aging, memory enhancing, anti-tumor, anti- arthritic, cytotoxic, and chemo-preventive (Table 3). These bioactivities have been discussed in the following sections, with brief information presented about their mechanisms.

### 8.1. Anti-Oxidant Properties

Antioxidants have been documented to prevent oxidative damage from free radicals responsible for a number of human disorders such as arthrosclerosis, diabetes mellitus, hypertension, arthritis, Alzheimer’s disease, ischemia gastritis and AIDS [80,109]. The antioxidant properties of Bacopa can offer protection against free radical damage in cardiovascular disease and certain types of cancer [110]. Reportedly, bacosides scavenge free radicals such as superoxides, peroxides and hydroxyl radicals.

Antioxidant effects of alcoholic and hexane extract of *B. monnieri* on lipid peroxidation by cumene hydroperoxide and ferrous sulphate is reported in rat homogenate liver [111]. Bacosides were found to have antioxidant activity in the hippocampus, frontal cortex and striatum on the basis of animal studies [112] and to modulate the expression of certain enzymes involved in generation and scavenging of reactive oxygen species in the brain and demonstrated that bacoside A3 had an inhibitory effect on superoxides released from polymorphonuclear cells in a nitroblue tetrazolium assay in the hydroalcoholic extract of the whole plant [113]. Sumathy et al. [114] reported the hepatoprotective activity of its orally administered alcoholic extract on the liver antioxidant status of morphine-treated rats. The defensive function of methanolic extract in rat astrocyte culture against the toxicity caused by the NO donor (S-nitroso-Nacetyl-penicillamine, SNAP), thus preventing DNA damage [2]. Janani et al. [104] reported the neuroprotective effect of the herb against aluminum triggered oxidative stress in the rat brain hippocampus. The free radical scavenging activity of the plant’s methanolic extract provided protection against DNA damage in non-immortalized fibroblasts human [115].

### 8.2. Anti-Cancer/Cytotoxic Properties

The ethanolic extract of *B. monnieri* contains bacoside A and B, betulinic acid, brahmine, and cucurbitacins, among which cucurbitacins have strong anti-tumorigenic and anti-proliferative activity by inducing cell cycle arrest at the G2/M phase and formation of multiplied cells. Mallick et al. [116] reported the cytotoxic activity of ethanolic extract of dichloromethane (DCM) fraction of *B. monnieri* on two different cell lines such as MCF-7 and MDA-MB 231 due to the presence of cucuebitacins and betulinic acid in DCM fraction. *B. monnieri* extracts induces cell death by apoptosis in murine sarcoma-180 cell culture [98]. Two new dammarane glycosides, the 20-deoxy derivatives of pseudojujubogenin along with eight novel compounds were isolated and tested them for cytotoxic, antileishmanial, anti-inflammatory, antimalarial activities. Some of these compounds demonstrated mild to moderate activity against non-cancerous kidney cell lines [117]. In addition, compared to bacoside B fraction, bacoside A fraction and its individual components were found to be more active [118]. *B. monnieri* shows curative and protective effects on gastric ulcers due to its improved mucosal activity in brine shrimp lethality assay (an assay that is predictive of potential anticancer activity) [119,120].

### 8.3. Anti-Inflammatory Properties

*B. monnieri* effectively suppressed experimentally induced inflammatory reaction effect by inhibiting the synthesis of prostaglandin and partly by stabilizing lysosomal membranes and did not cause gastric irritation at anti-inflammatory doses [121,122]. Methanol extract of the whole plant produced significant writhing inhibition in acetic acid induced writhing in mice at the oral dose of 250 and 500 mg/kg (*P* < 0.001) comparable to 25 mg/kg diclofenac sodium [123]. The anti-inflammatory effects of the different extracts of *B. monnieri* on carrageenan induced edema in the hind paws of rats were investigated. The methanol extract and aqueous fractions (100 mg/kg) showed a significant decrease in the volume of edema paw, whereas hexane extracts and petroleum ether didn’t reduce inflammation [124].

### 8.4. Gastrointestinal Properties

The antidiarrheal effect on castor oil induced diarrhea in mice was shown in the ethanol extract of the whole plant of *B. monnieri*. It increased mean latent period and significantly decreased the frequency of defecation at the oral dose of 500 mg/kg comparable to 50 mg/kg loperamide [123]. It shows curative and protective effect on gastric ulcers due to its effect on mucosal defensive factors like enhanced mucin secretion, mucosal glycoprotein and decreased cell shedding rather than on offensive factors such as acid and pepsin [125]. The *Bacopa* extract standardized for bacoside-A was evaluated in five models of gastric ulcers in rats for its prophylactic and healing effects. Bacopa extract significantly healed an ulcer induced by acetic acid at a dosage of 20 mg/kg for 10 days, decreased mucosal exfoliation, and significantly strengthened the mucosal barrier [126].

### 8.5. Anti-Alzheimer’s Properties

Alzheimer’s disease (AD) is a progressive neurodegenerative disorder of the brain that is characterized by impairment of memory and eventually by disturbances in reasoning, planning, language, and perception [127]. Currently, there is no drug or therapy available as a definite solution for the treatment of AD except two main classes of drugs, namely acetyl cholin esterase inhibitors (AChEI) for the treatment of mild to moderate AD and glutamate modulators only for moderate to severe AD [128]. One of the most important approaches for the treatment of AD involves the enhancement of acetylcholine level in the brain using AchEI inhibitors. The ethanol extract of *B. monnieri* contain bacoside A, which has been used for memory and intellectual improvement. Clinical trial reported that when 300 mg of standardized *B. monnieri* extract was taken orally twice a day for six months results in improvement in cognitive functions of patients suffering from AD [129]. The in vivo study of ethanol extract of *B. monnieri* was done in male albino rat (225–250 g) in which the oral dose of (100 mg/kg body weight) ethanol extracts given over the 15 days and it inhibits the function of acetylcholinesterase differentially in various brain regions viz. cerebral cortex (51.6%) > CEREBELLUM (51%) > PONS (44%) > THALAMUS (41.6%) > HIPPOCAMPUS (38.1%) > BRAIN stem (34.3%) > STRIATUM (24.9%) [100].

## 9. Conclusions

It is evident from the review of literature that considerable research has been conducted on the in vitro propagation of Brahmi (*B. monnieri*) plants and a judicial choice of protocol is required for the mass clonal propagation for cost effective commercialization. The multiplication of shoots for the clonal propagation of *B. monnieri* has been reported using pre-existing meristems such as nodes and shoot tips derived from both ex vitro and in vitro grown plants. Among the two types of explants used, nodes have been found to be the better choice of explants for in vitro shoot multiplication. Further, on the basis of research reviewed, it can be concluded that both internode and leaf explants derived from ex vitro plants show a significantly higher potential for micropropagation (155–324 shoots/explant) than similar explants derived from in vitro plants (Table 1).

Although the morphogenic potential of *B. monnieri* explants appears to be very high, as evident in most of studies, the choice of explants and regeneration protocol will be important for maintaining genetic stability avoiding somaclonal variations. Hence, it is suggested that both axillary bud multiplication and adventitious bud induction using leaf explants (without intervening callus phase) can be explant of choice using cytokinins like BA and TDZ as reported. A novel technique for the production of true to type plants has been reported by Faisal et al. [66] using TDZ pulse treated nodal explants and the protocol suggested ensures the genetic stability of micropropagated plants. However regeneration rates in phytohormone supplemented medium is more, as expected and as reported by large number of researchers (Table 1).Another interesting point observed in the in vitro derived plant is the expression of totipotency of excised leaf and stem explants from in vitro plants in PGR free medium and may be used as a model system for study on causes of morphogenesis in vitro [65]. Shoot culture in bioreactors is a promising possibility for obtaining higher biomass and bacoside yield, and could be a commercially viable future [56]. Recently, it has been reported that bioactive compounds in *B. monnieri* in vitro cultures can be enhanced by feeding precursors and LED light exposure. Such approaches may be used for shoot cultures in bioreactors in vitro [130].

The medicinal and pharmacological importance of bacopa is increasing daily. *B. monnieri* shows massive potential to relieve various neuropharmacological, inflammation, depressions, and other disorders. For future, however, voluminous research is required to verify its efficacy for various disorders. The ethanolic and methanolic extract of bacopa plays a crucial role in treating human diseases at varying concentrations. Bacoside A is the extensive chemical agent responsible for therapeutic effects identified across various research models. Nevertheless, further studies are required to determine the targeted activity of the bioactive compounds present in the isolated bacoside fraction of BM. The antioxidant activity of bacopa may be useful to treat human pathologies in which free radical production plays a crucial role, which requires further research. Biomedical study of bacopa is still in its formative years, but preliminary results like those depicted in this review can definitely open the floodgates to young researchers.

## Figures and Tables

**Table 1:** In vitro propagation of *Bacopa monnieri* (L.) Wettst. (Chronological listing).

Explant-Source/Type	Culture Medium, PGRs and Additives	Remarks, Experimental Outcome and Maximum Productivity, Acclimatization etc.	References
Stem segments with nodes (20 mm) of *ex vitro* plants	MS + 0.2 mg L^−1^ NAA + 0.5 mg L^−1^ BA + 50 mg L^−1^ glutamine + 75–100 µM CuSO_4_ (SIM).	15.52 ± 2.77 shoots/nodal explant within 4-w of culture on SIM. At 100 µM ofCuSO_4_ maximum multiplication could be achieved and tolerant cultures could be raised.	[20]
Terminal shoots bearing 4–5 nodes from field-grown plants	MS + 2.0 mg L^−1^ BA (SIM); MS + 0.1 mg L^−1^ BA + 0.2 mg L^−1^ IAA (SELM). MS + 1.0 mg L^−1^ NAA (RIM). MS + 0.5 mg L^−1^ BA (SEIM; callus explants).	79 shoots/leaf explant, 20 shoots/node and 26 shoots/internode formed on SIM within 4-w. 100% of the shoots rooted on RIM. SE developed after 4-w of culture on SEIM. Histological analysis of the calli revealed typical heart-shaped and cotyledonary stage somatic embryos. Plantlets acclimatized in sterilized soilrite with 95% survival rate.	[21]
Stem segments with nodes (20 mm) of *ex vitro* plants	MS + 0.2 mg L^−1^ NAA + 0.5 mg L^−1^ BA + 50 mg L^−1^ glutamine + 400 µM ZnSO_4_	This study was on effect of ZnSO_4_ on the morphogenic response. 24 shoots/culture formed within 4-w on SIM containing 400 µM ZnSO_4_. 100% of shoots rooted. Proline and protein accumulated as a sequel to zinc stress.	[22]
Explants from field-grown plants	MS + 2 µMBA (SIM).	A thick mat of shoot buds formed on 90–100% of the explant surface after 3–4 weeks on SIM. The potency was expressed as percentage of the surface area of the explants showing organogenesis.	[23]
Nodal explants (1.0 cm) of field grown plants	MS + 6.8 μM TDZ (SIM)/+2.2 μM BA (SMM)/4.9 μM IBA (RIM).	92 shoot buds/leaf explant, 42 adventitious shoot buds/node and 28 adventitious shoot buds/internode after 7-w of culture on SIM. 129 shoot buds/leaf explant on SMM after 3 subcultures (each with 4 weeks duration). 100% of shoots rooted on RIM within 2-w. Acclimatization in sterilized soilrite with 100% survival rate.	[24]
Nodal segments (~0.5 cm) with single axillary buds of field grown plants	MS + 1.0 mg L^–1^ BA + 1.0 mg L^–1^ IBA (SIM, SMM).	18.35 ± 2.15 shoots/culture formed within 4-w. Regenerated shoots successfully rooted. HPLC and LC-MS analysis identified Bacoside A3 and A2. The total bacosides ranged between 1.76–2.70% on dry weight basis. Acclimatization in sand: garden soil potting mixture (1:1) with 90% survival rate.	[25]
Axillary nodes, young leaves and internodes of 3-m-old *ex vitro* plants	Liquid MS + 1.1 μM BA + 0.2 μM IAA (SIM, SMM, RIM; leaf explant).	110 shoots/leaf explant formed within 3-w on SIM while shoot induction was very low from nodal explants (7–8 shoots/node). Acclimatization in sand, soil and farmyard manure (1:1:1) with 98% survival rate. HPLC revealed a phytochemical profile similar to that of the market sample and mother plants.	[26]
Nodal segments (5–6 nodes; 7–8 cm) of *ex vitro* plants	MS + 2.0 mg L^−1^ BA (SIM). MS + IBA (0.5–2.0 mg L^−1^) (RIM).	Nodal explants formed 9.4 shoots/explant after 7-d. on SIM while leaf explants formed 4.3 shoots/explants after 15-d.	[10]
Nodal explants internodes and leaf of 4-week-old in vitro grown shoots	MS + 300 mg L^−1^ BVN (SIM)/+0.2 mg L^−1^ IAA + 0.1 mg L^−1^ BA (SELM)/	100% of explants formed shoots with 98 shoots/internode explant, 81 shoots/leaf explant and 21 shoots/nodal explant on SIM within 4-w. Optimum shoot growth in SELM. Acclimatization in sterilized soilrite with 85% survival rate in field.	[27]
Tender leaves of 2-m old greenhouse plants	MS + 0.5 mg L^−1^ 2,4-D (CIM, SEIM).	Embryogenic callus after 60 d with 42 embryoids/callus on SEIM, clearly identified globular, cordate, torpedo embryos. 28 rooted plantlets/embryoid formed on SEGM within 45-d. 98% survival rate.	[28]
Internodes (2.5 cm) of 2–3-month-old ex vitro plants	MS + 1.0 mg L^−1^ BA + 0.5 mg L^−1^ Kn (SIM). Liquid MS + 1.5 mg L^−1^ NAA (RIM).	18 shoots/explant formed and an average of 324 shoots/explant were generated after 3 subcultures. 12 ± 1.73 roots/shoot formed on RIM. Plantlets acclimatized in garden soil: vermiculite: sand (1:1:1) with 100% survival rate in field.	[29]
Shoot apex and nodes (1–1.2 cm) of young greenhouse plants	MS + 5.0 mg L^−1^ BA + 0.2% (*w/v*) NaCl/10% Mannitol.	20 shoots/culture without root formed after 15-d under salt stress. Shoots with 4–5 roots/explant formed after 15-d under drought stress.	[30]
Young leaves of field grown plants→2% NaOCl 5 min→SDW→0.1% HgCl_2_ 2 min	MS + 2 mg L^−1^ Kn (SIM). Liquid MS + 2 mg L^−1^ Kn (SMM). pH 5.8. 2% sucrose. 0.7% agar.	An optimum of 155 shoots proliferated/explant at the end of 8th week on SMM. HPLC analysis revealed bacoside A contents were highest in case of shoots regenerated in SMM (11.92 mg g^−1^ DW).	[31]
Nodal explants (1.5 cm) of 2–3-m-old *ex vitro* plants	MS + *Aulosira fertilissima* extract + 0.44 gm/L CaCl_2_ (SIM). MS + *A. fertilissima* extract + 1.0 mg L^−1^ Kn + 0.44 gm/l CaCl_2_ (SMM, RIM). pH 5.8. 3% sucrose. 0.8% agar	20 shoots/nodal explant on SIM within 2 w. 56 shoots/explant formed on SMM after 4-w. 100% of shoots rooted with 15 ± 2.20 roots/shoot. Plantlets acclimatized in garden soil, mixed with vermiculite and sand (1:1:1) and successfully transferred to the field with 100% survival rate.	[32]
Leaf, internode shoot buds of *ex vitro* plants	MS + 1.5 mg L^−1^ TDZ + 0.5 mg L^−1^ NAA (SIM). MS + 0.5 mg L^−1^ BA (SMM). ½ MS + 1.0 mg L^−1^ IBA + 0.5 mg L^−1^ Phloroglucinol (RIM).	56 shoots/leaf explant and 49 shoots/internode explant after 3-w. 135 shoots/leaf explant and 112 shoots/internode explant on SMM after 4-w. 16 roots/shoot on RIM after 4-w. Acclimatization in sterilized vermicompost supplied with diluted MS basal salts with a 100% survival rate. RAPD profile confirmed clonal fidelity.	[33]
Apical portions of healthy twigs of *ex vitro* plants bearing leaves up to the fourth node were excised	MS + 6 μM BA + 3% sucrose (SIM). Liquid ½ MS + 2 μM IBA + 1% sucrose (RIM)	63 shoots/explant after 8-w. Rooting of micro shoots within 2-w. Plantlets acclimatized in sand: soil (3:1) mixture under greenhouse conditions.	[34]
Leaves (0.75 cm^2^) of *ex vitro* plants	Liquid MS + 5 µM BA + 100 µM pyruvic acid (SIM). MS + 5µM NAA + 1 µM 2,4-D + 100 µM pyruvic acid	100 µM pyruvic acid effectively enhanced the production of bacoside-A in shoot as well as callus biomass. The bacoside-A content in in vitro raised shoot biomass was 1.2 times higher as compared to shoot biomass of naturally grown plants.	[35]
Nodal segments of *ex vitro* plants	MS + 0.1 mg L^−1^ BA (SIM, SMM). MS + 0.15 mg L^−1^ IBA (RIM).	100% of cultures showed axillary bud break; 41 shoots/explant after 4-w. 100% shoots formed roots with 24 roots/shoot within 3–4 w. Plantlets acclimatized in a mixture of sand, farmyard manure and soil (1:1:1) irrigated with ½ MS medium and finally shifted to shade house.	[36]
Leaf explants of *ex vitro* plants	MS + 2.0 mg L^−1^ BA + 0.5 mg L^−1^ NAA + 3% sucrose (CIM, SIM). ½ MS + 2.0 mg L^−1^ IAA + 2% sucrose (RIM).	61% callus induced formed shoots with 16 shoots/callus after 5-w. 6 roots/shoot formed on RIM after 3-w. Acclimatization in sterilized sand: soil: dry powdered cow dung (1:1:1) with mild irrigation at 2-day interval and supplied with ¼ strength MS inorganic solution twice a week and transferred to filed with 86% survival rate.	[37]
Leaf and stem of *ex vitro* plants	MS + 2.5 mg L^−1^ BA + 0.01 mg L^−1^ IAA (SIM). Liquid MS + 2.5 mg L^−1^ BA + 0.01 mg L^−1^ IAA (SIM; node) in bioreactor.	20 nodal explant/40 mL medium was optimal for high explant response. Maximum growth index (10.0) was recorded in bioreactor producing ~2000 shoots/L with 16.5 g/L DW. The total phenolic content and antioxidant capacity of in vitro grown plants was higher to that recorded for in vivo plants.	[38]
Nodes of *ex vitro* plants	MS + 0.25 mg L^−1^ 2,4-D + 0.5 mg L^−1^ Kn (CIM; leaf petiole).	Plantlets acclimatized in culture bottles ¼th filled with Soilrite composition (soil: sand: peat moss) and irrigated with ¼ MS salt solution and then shifted to misthouse with 90% survival rate.	[39]
Nodes, shoot tip of 3-month-old *ex vitro* plants	MS + BA + Kn + NAA (each 1.0 mg L^−1^ SIM).MS + 1.0 mg L^−1^ IBA + 0.5 mg L^−1^ NAA (RIM).	100% of explants formed shoots after 4-w and rooted on RIM with after 4-w. Acclimatization in sterile soil and perlite (1:1) with 96% survival rate in field. Phytochemical profile similar to that of the field grown plants.	[40]
Leaf and internodes of *ex vitro* plants	MS + 2.0 mg L^−1^ BA (SIM). MS + 0.5 mg L^−1^ GA3 (SMM). ½ MS + 2.0 mg L^−1^ IBA (RIM).	Shoot organogenesis with 104 shoots/leaf explant and 89shoots/internode explant on SIM. 100% of shoots rooted with 57 roots/shoot and on RIM. Acclimatization in sterile vermiculate: sand: soil (1:2:2) with 90% survival rate in greenhouse.	[41]
Shoot tips (0.7–1.2 cm) of field grown plants	MS + 1.0 mg L^−1^ BA.	86% of encapsulated nodal explants germinated into plantlets after 6-8 w. Acclimatization in a potting mix of sand: soil (1:1) and finally transferred to net house.	[42]
Shoots of *ex vitro* plants	MS + TMP + BVN (each 200 mg dm^−3^; SIM). MS + 0.2 mg dm^−3^ IAA + 0.1 mg dm^−3^ BA (SELM). MS + 0.5 mg dm^−3^ IBA (RIM).	135 shoot buds/internode explant, 90 shoot buds/leaf explant and 50 axillary shoots/nodal explant on SIM. Optimum shoot growth on SELM. 90% of elongated shoots rooted on RIM. Acclimatization in soilrite with 90% survival rate in field.	[43]
Axillary nodes, young leaves and shoot tips of 3-m-old ex vitro plants	70% strength MS + 3 mg L^−1^ Kn + 0.5 mg L^−1^ IBA (SIM).	100% of explants formed shoots (low multiplication rate) and rooted plantlets acclimatized in sand, soil and farmyard manure in the ratio of 1:1:1.	[44]
Shoots (5–6 cm) with node explants of *ex vitro* plants	Cyanobacterial medium + 2 mg L^−1^ Kn (SIM).	80% of explants formed shoots with very low multiplication rate.	[45]
Nodes, internodes, shoot tips and leaves of green house plants	MS + 1.0 mg L^−1^ BA + 0.5 mg L^−1^ NAA (SMM). MS + 0.25 mg L^−1^ IBA (RIM).	85% nodal explants showed multiple shoot formation; 42 shoots/explant on SMM. 86% shoots rooted. Acclimatization in sand: compost mixture (1:2) in the greenhouse and with 70–80% survival rate in field.	[46]
Twigs with 4–5 nodes with attached leaves of outdoor plants	MS + BA + NAA (each 0.25 mg L^−1^; SIM). MS + 0.25 mg L^−1^ IBA (RIM).	100% of explants showed callus induction and shoot regeneration within 1 w. 23 shoots/internode and 21 shoots/leaf explant after 6 w. 100% shoots rooted in RIM and survived (100%) after acclimatization.	[47]
Nodes of *ex vitro* plants	MS + 1.0 mg L^−1^ BA + 3% sucrose (SIM, SMM). ½ MS *+* 1.0 mg L^−1^ IBA + 2% sucrose (RIM).	5.0 axillary shoots/explant on SIM after 4 w. 20 shoots/induced axillary shoot formed after 4 w. 10 roots/shoot formed on RIM after 4 w. Acclimatization in a mixture of soil: sand: manure (1:1:1) with 100% survival rate in field.	[48]
Cotyledonary and epicotyledonary nodes obtained from axenic 15-d-old seedlings	MS + 0.5 mg L^−1^ Kn + 1.0 mg L^−1^ BA (SMM). ¼ MS + 2.0 mg L^−1^ IBA + 1.0% sucrose (RIM).	95% of explants formed shoots and rooted. Mulitiplication rate low. Acclimatization in soilrite with 90% survival rate when transferred to mist house.	[49]
Young and juvenile nodal segments of *ex vitro* plants	MS + 1.0 mg L^−1^ BA (SIM). MS + 1.5 mg L^−1^ BA (SMM, SELM). ½ MS + 1.0 mg L^−1^ IBA (RIM).	57% of explants formed shoots with 2.64 shoots/explant within 20 d. 90% of shoots rooted within 10 d. Plantlets hardened in vitro on liquid ¼ MS + 2% sucrose. Acclimatization in poly bags containing a mixture of soil and sand (1:1).	[50]
Twigs with 4–5 nodes of *ex vitro* plants	MS + 2.0 mg L^−1^ BA (SIM). MS + (0.25, 0.5, 1.0) mg L^−1^ IBA (RIM).	Direct adventitious shoot regeneration within 7–8 days of culture; 26 shoots/explant after 8 w.100% rooting on RIM after 2-w. Acclimatization in pots containing organic matter or jars containing water (pH 7).	[51]
Young leaves, nodes and internodes of *ex vitro* plants	MS + 2.0 mg L^−1^ Kn (SIM, RIM).	72 shoots/leaf explant, 67 shoots/internode explant and 64 shoots/node explant after 8 w. Acclimatization in sterilized soil-rite with 95% survival rate.	[52]
Leaf of *ex vitro* plant	MS + 2 mg L^−1^ Kn (SIM; leaf). MS (SELM, RIM). MS + 1 mg L^−1^ 2,4-D (CIM).	126 shoots/leaf explant formed after 45-d. Micro shoots elongated and rooted on RIM in 15-d. Acclimatization in soil rite (mixture of coco brick, cocopeat perlite and vermiculite). Detection of Bacopaside I and II in micro shoots by HPLC.	[53]
Nodal segments (1.0–1.5 cm) of *ex vitro* plants	MS + 3.0 mg L^−1^ BA (SIM)/+ 1.0 mg L^−1^ + GA3 (SMM, SELM). ½ MS + 0.2 mg L^−1^ IBA (RIM).	Shoot bud induction after 4-w. 114 shoots/explant with shoot length 6.4 cm after 3-w of sub-culture. Shoots rooted with 10 roots/shoot on RIM after 2-w. Acclimatization in plastic pots containing garden soil with 100% survival rate.	[54]
Twigs with 3–4 nodes of *ex vitro* plants	MS + 5.0 μg/mL BA (SIM, SMM). MS + NAA/IAA + 2% sucrose (RIM).	Low rate of multiplication. Acclimatization in soil rite: red sand: garden soil (3:2:1).	[55]
Shoot cultures from nodal segments	1.5 L liquid MS + 1 mg L^−1^ BAP (SIM).	The present work reports bioreactor based micropropagation and bacoside biosynthesis.443 shoots with 5.84 growth index in terms of dry wt. was recorded after 4-w. Bacoside production in shoot cultures of ALB system was ~1.75-fold higher.	[56]
Axenic cultures→leaf and internode→TCL	MS + 10.0 µM BA (SIM). Liquid MS + 1% sucrose (SELM). MS + 5.0 µM IBA (RIM).	59 adventitious shoot buds/leaf tTCL explant 33 adventitious shoot buds/internode tTCL explant on SIM. 100% of shoots rooted on RIM. Acclimatization in plastic pots containing potting mixture under mist chamber and finally transferred to green house.	[57]
Shoot tips (≤5 mm) from *in vitro* shoot cultures	2.5% Na- alginate in MS + 3% sucrose→100 mM CaCl_2_.2H_2_O (encapsulation).	93% of encapsulated shoot tips showed regrowth with 10 shoots/explant after 6 months of storage at 4 °C. 100% of shoots showed rooting. Acclimatization in pot mixture of peat-moss and sand (1:1) with 93% survival rate in greenhouse.	[58]
Stems (4–6 cm) with 4–5 nodes and leaves	MS + 0.25 mg L^−1^ BA (SIM). MS + 0.25–1.0 mg L^−1^ IBA (RIM).	Direct shoot induction within 8-10 d from leaf explants; 27 shoots/upper half of explant on SIM under W LED lighting system after 8-w. 100% of shoots rooted on RIM after 4-w. Acclimatization in aquarium with sand and tap water (pH ~8.0).	[59]
Axenic shoot cultures→nodal explants	MS + 12.5 μM BA + 1 μM 2,4-D + 50 mM sucrose (SIM). MS + 12.5 μM BA + 1 μM 2,4-D + 250 mM sucrose (SEIM). ½ MS (SEGM).	100% of leaf explants differentiated shoot buds on SIM within 30-d 100% of SEs formed complete plantlets on SEGM after 6-w. Stages of direct SE differentiation confirmed by SEM. 41% of encapsulated SEs stored at 25 °C produced complete plantlets within 20 d of culture on MS medium.	[60]
Nodal segments from *ex vitro* plants	MS + 30% *Sargassum wightii* liquid extracts (SIM, RIM).	This study suggests that seaweed liquid extract can be used as substitute for PGR for in vitro propagation.25 shoots/explant formed with increased biomass after 3rd subculture. Acclimatization in autoclaved soil and vermiculate (1:1).	[61]
Nodal explants	Liquid MS + 1 mg L^−1^ Kn (SIM, RIM). Subcultures 7-d.	36–75 rooted shoots along with adventitious shoots were formed after 7-d in SIM. Acclimatization in sterilized garden soil in a shade house for one week. Aqueous extracts of acclimatized plants showed 10-fold higher antioxidant capacity than in vitro liquid cultured plantlets.	[62]
Shoot tips (8–10 mm) of *ex vitro* plants	MS + 1.5 mg L^−1^ BA + 2.0 mM Spermidine (SIM, SELM). ½ MS (RIM).	123 adventitious shoot buds/explant with 34.9 elongated shoots/explants were formed after 12-d. 13 roots/shoot formed on RIM within 18-d. Acclimatized with 96.7% survival rate in field.	[63]
Shoot tips of *ex vitro* plants	MS + 60% *G. salicornia* extract (SIM, SMM). ½ MS + 0.1 mg L^−1^ IAA (RIM). pH 5. 75. 3% sucrose. 0.8% Agar–agar type-1, 0.2% CleriGel	94% encapsulated shoot tip explants multiplied resulting 92 shoots/explant after 1-w. 85% explants showed multiple shoot induction with 145 shoots/encapsulated explants after 2-w. 84% shoots formed 54 roots/shoot within 1-win RIM. Acclimatization in sterile Soilrite (Keltech, Bengaluru, India): peat (1:1) in growth chamber with 100% survival rate in green house. RAPD analysis of in vitro regenerated plants and mother plants showed genetic similarity.	[64]
Shoot tips of *ex vitro* plants	PGR free MS medium	Explants excised from different positions showed variable frequency of direct shoot organogenesis in unsupplemented MS medium with optimum of 8 shoot buds/leaf and 15/internode explant within 4-w. 100% of micro shoots (5–6 cm long) rooted within 2-w of culture. Acclimatized plants flowered within 3 m.	[65]
Nodal explants	Liquid MS + 20 uM TDZ	43 shoots per explant were obtained after 8-w of culture. Microshoots were rooted in growth regulator free media. Acclimatized with 97% survival ex vito.	[66]

2,4-D 2,4-dichlorophenoxyacetic acid; BA N6-benzyladenine (BA is used throughout even though BAP (6-benzylamino purine) may have been used in the original; BVN Bavistin Methyl benzimidozole carbamate; CIM Callus induction medium; d day(s); GA_3_ Gibberellic acid; HgCl_2_ Mercury chloride; HPLC High performance liquid chromatography; IAA Indole-3-acetic acid; IBA Indole-3-butyric acid; Kn Kinetin (6-furfuryl aminopurine); LED Light emitting diodes; m Month(s); MS Murashige and Skoog medium [67]; NAA α-naphthaleneacetic acid; NaOCl Sodium hypochlorite; PGR(s) Plant growth regulator(s); RAPD Random amplified polymorphic DNA; RIM Root induction medium; SDW Sterilized (by autoclaving) distilled water; SE Somatic embryo; SEM Scanning electron microscope; SEGM Somatic embryo germination medium; SEIM Somatic embryo induction medium; SELM Shoot elongation medium; SIM Shoot induction medium; SMM Shoot multiplication medium; TDZ Thidiazuron (N-phenyl-N’- 1,2,3-thiadiazol-5-ylurea); TMP Trimethoprim (2, 4 di-amino-5-(3, 4, 5-trimethoxybenzyl) pyrimidine; w week(s).

**Table 2:** Characterization and isolation of Saponins from the alcoholic extract reported from *B. monnieri.*

S. No	Compounds	Derivatives	Reference
**Jujubogenin derivatives**			
1	Bacoside A_1_	3-O-[α-L-arabinofuranosyl(1→3)]-α-L-arabinopyranoside	[85]
2	Bacoside A_3_	3-O-α-L-arabinofuranosyl-(1→2)-[β-D-glucopyranosyl-(1→3)]-β-D-glucopyranoside	[86]
3	Bacopaside III	3-O-α-L-arabinofuranosyl-(1→2)-β-D-glucopyranosyl	[87]
4	Bacopaside IX	3-O-{β-D-glucopyranosyl(1→4)[α-L-arabinofuranosyl-(1→2)]-β-D-glucopyranosyl}-20-O-α-L-arabinofuranosyl	[88]
5	Bacopaside X	[α-L-arabinofuranosyl-(1→2)-[β-D-glucopyranosyl-(1→3)]-α-L-arabinofuranosyl]	[89]
6	Bacopaside N1	[β-D-glucopyranosyl-(1→3)]-β-D-glucopyranosyl]	
7	Bacopaside IV	3-O-β-D-glucopyranosyl-(1→3)-α-L-arabinofuranosyl	[87]
8	Bacopasaponin A	3,20-di-O-α-L-arabinopyranoside	[90,91]
9	Bacopasaponin E	3-O-α-L-arabinofuranosyl-(1→2)-[β-D-glucopyranosyl-(1→3)]-α-L-arabinopyranoside,20-O-α-L-arabinopyranoside	[92]
10	Bacopasaponin F	3-O- α-L-arabinofuranosyl-(1→2)-[β-D-glucopyranosyl-(1→3)]-β-D-glucopyranoside,20-O-α-L-arabinopyranoside	
11	Bacopasaponin G	3-O-[α-L-arabinofuranosyl-(1→2)]-α-L-arabinopyranoside	[79]
**Pseudojujupogenin derivatives**			
1	Bacoside A_2_	3-O-α-L-arabinofuranosyl-(1→5)-[α-L-arabinofuranosyl-(1→6)]-α-D-glucofuranoside	[93]
2	Bacopaside N2	[β-D-glucopyranosyl-(1→3)-β-D-glucopyranosyl]	[89]
3	Bacopaside III	3-O-[6-O-sulfonyl-β-D-glucopyranosyl-(1→3)]-α-L-arabinopyranoside	[79]
4	Bacopaside II	3-O-α-L-arabinofuranosyl-(1→2)-[β-D-glucopyranosyl-(1→3)]-β-D-glucofuranoside	[87,94]
5	Bacopaside I, Bacopaside V	3-O-α-L-arabinofuranosyl-(1→2)-[6-O-sulfonyl-β-D-glucopyranosyl-(1→3)]-α-L-arabinopyranoside, 3-O-β-D-glucopyranosyl-(1→3)]-α-L-arabinofuranosyl	
6	Bacopasaponin B	3-O-[α-L-arabinofuranosyl-(1→2)]-α-L-arabinopyranoside	[90,91]
7	Bacopasaponin D	3-O-[α-L-arabinofuranosyl-(1→2)]-β-D-glucopyranoside	
8	Bacopasaponin C	3-O-[α-L-arabinofuranosyl-(1→2)]-β-D-glucopyranosyl-(1→3)]-α-L-arabinopyranoside	
9	Bacopasaponin H	3-O-[α-L-arabinofuranosyl]	[95]
10	Bacopaside XI	3-O-[β-D-arabinofuranosyl-(1→3)]-6-O-sulfonyl-β-D-glucopyranosyl	[96]
11	Bacopaside XII	3-O-{β-D-glucopyranosyl(1→3)[β-D-arabinofuranosyl(1→2)]-β-D-glucopyranosyl}-20-O-β-D-arabinopyranosyl	

**Table 3:** Pharmacological activities of *B. monnieri* (L.) and its active constituents.

Compounds/Extracts	Pharmacological Activity	Experimental Model	Dosage/Concentration/Period of Administration	References
*B. monnieri* methanol extract of the whole plant; bacoside E; bacopaside VII	Anti-tumor	Human tumor cell lines MDA-MB-231, SHG-44, HCT-8, A-549 and PC-3M	50 μmol/kg for 7days	[97]
*B. monnieri* ethanol extract	Apoptopic/cytotoxic	In mouse S-180 cells	550 μg/mL for 48 h	[98]
	Improving learning & memory	Serotonergic system of postnatal rats	40 mg/kg for 15 days	[99]
	Anti- Alzheimer’s	Adult male rats	100 mg/kg for 15 days	[100]
Standardized *B. monnieri* extract	Memory enhancer	Human	125 mg of SBME or placebo twice a day for 16 weeks	[101]
*B. monnieri* methanol extract	Anti-inflammatory	Carrageenan-induced rat paw edema	100 mg/kg for 5 h	[102]
Bacoside A	Anti- apoptosis	Adult male albino rats of Wistar strain	10 mg/kg/day for 12 weeks	[103]
	Hepatoprotective	Rats induced with N- nitrosodiethylamine	15 mg/kg/day for 14 days	[104]
	Neuroprotective	Rat brain exposed to cigarette smoke	10 mg/kg/day for 12 weeks	[105]
	Anti-oxidant	Rat brain exposed to cigarette smoke	10 mg/kg/day for 12 weeks	[106]
	Anti-epileptic	Pilocarpine-induced epileptic rats	150 mg/kg/day for 15 days	[18]
*B. monnieri* n- butanol extract	Acquisition and expression of morphine tolerance	Mice	15 mg/kg for 7 days	[107]
*B. monnieri* alcohol extract	Cognitive function enhancer and neuroprotective	Male Wister rats induced by ethylcholine aziridinium ion (AF64A)	40 mg/kg for two weeks	[108]

## References

[1] Gohil K.J., Patel J.A. (2010). A review on *Bacopa monniera*: Current research and future prospects. Int. J. Green Pharm..

[2] Russo A., Borrelli F. (2005). *Bacopa monniera*, a reputed nootropic plant: An overview. Phytomedicine.

[3] Majumdar S., Basu A., Paul P., Halder M., Jha S., Ramawat K., Mérillon J.M. (2013). Bacosides and Neuroprotection. Natural Products.

[4] Aguiar S., Borowski T. (2013). Neuropharmacological review of the nootropic herb *Bacopa monnieri*. Rejuvenation Res..

[5] Rajan K.E., Preethi J., Singh H.K. (2015). Molecular and functional characterization of *Bacopa monniera*: A retrospective review. Evid. Based Complement. Altern. Med..

[6] Jyoti A., Sharma D. (2006). Neuroprotective role of *Bacopa monniera* extract against aluminium-induced oxidative stress in the hippocampus of rat brain. NeuroToxicology.

[7] Jyoti A., Sethi P., Sharma D. (2007). Bacopa monniera prevents from aluminium neurotoxicity in the cerebral cortex of rat brain. J. Ethnopharmacol..

[8] Kamkaew N., Scholfield C.N., Ingkaninan K., Taepavarapruk N., Chootip K. (2013). *Bacopa monnieri* increases cerebral blood flow in rat independent of blood pressure. Phytother. Res..

[9] Rastogi M., Ojha R.P., Prabu P.C., Devi B.P., Agrawal A., Dubey G.P. (2012). Prevention of age-associated neurodegeneration and promotion of healthy brain ageing in female Wistar rats by long term use of bacosides. Biogerontology.

[10] Mohapatra H.P., Rath S.P. (2005). In vitro studies of *Bacopa monnieri*—An important medicinal plant with reference to its biochemical variations. Indian J. Exp. Biol..

[11] Sharma N., Satsangi R., Pandey R., Devi S., Vimala S. (2007). In vitro clonal propagation and medium term conservation of Brahmi (*Bacopa monnieri*). J. Plant Biochem. Biotechnol..

[12] Bhattacharya S.K., Kumar A., Ghosal S., Siva Sankar D.V. (1999). Effect *of Bacopa monniera* on animal models of Alzheimer’s disease and perturbed central cholinergic markers of cognition inrats. Molecular Aspects of Asian Medicines.

[13] Deb D.D., Kapoor D., Dighe D.P., Padmaja D., Anand M.S., D’Souza P., Deepak M., Murali B., Agarwal A. (2008). In vitro safety evaluation and anticlastogenic effect of BacoMind on human lymphocytes. Biomed. Environ. Sci..

[14] Sairam K., Dorababu M., Goel R.K., Bhattacharya S.K. (2002). Antidepressant activity of standardized extract of *Bacopa monniera* in experimental models of depression in rats. Phytomedicine.

[15] Sairam L., Rao C., Babu M., Goel R.K. (2001). Prophylactic and curative effects of *Bacopa monniera* in gastric ulcer models. Phytomedicine.

[16] Bhatia G., Palit G., Pal R., Singh S., Singh H.K. (2003). Adaptogenic effect of *Bacopa monniera* (Brahmi). Pharmacol. Biochem. Behav..

[17] Khan R., Krishnakumar A., Paulose C.S. (2008). Decreased glutamate receptor binding and NMDA R1 gene expression in hippocampus of pilocarpine-induced epileptic rats: Neuroprotective role of *Bacopa monnieri* extract. Epilepsy Behav..

[18] Mathew J., Paul J., Nandhu M.S., Paulose C.S. (2010). Increased excitability and metabolism in pilocarpine induced epileptic rats: Effect of *Bacopa monnieri*. Fitoterapia.

[19] Mathew J., Soman S., Sadanandan J., Paulose C.S. (2010). Decreased GABA receptor in the striatum and spatial recognition memory deficit in epileptic rats: Effect of *Bacopa monnieri* and bacoside-A. J. Ethnopharmacol..

[20] Ali G., Srivastava P.S., Iqbal M. (1998). Morphogenic response and proline content in *Bacopa monniera* cultures grown under copper stress. Plant Sci..

[21] Tiwari V., Singh B.D., Tiwari K.N. (1998). Shoot regeneration and somatic embryogenesis from different explants of Brahmi [*Bacopa monniera* (L.) Wettst.]. Plant Cell Rep..

[22] Ali G., Srivastava P.S., Iqbal M. (1999). Morphogenic and biochemical responses of *Bacopa monniera* cultures to zinc toxicity. Plant Sci..

[23] Shrivastava N., Rajani M. (1999). Multiple shoot regeneration and tissue culture studies on *Bacopa monnieri* (L.) Pennell. Plant Cell Rep..

[24] Tiwari V., Tiwari K.N., Singh B.D. (2001). Comparative studies of cytokinins on in vitro propagation of *Bacopa monniera*. Plant Cell Tiss. Organ Cult..

[25] Ahuja A., Gupta K.K., Khajuria R.K., Sharma A., Kumar A., Sharada M., Kaul M.K., Kumar A., Roy S. (2005). Production of bacoside by multiple shoot cultures and in vitro regenerated plantlets of selected cultivar of *Bacopa monnieri* (L.) Wettst. Plant Biotechnology & Its Applications in Tissue Culture.

[26] Binita B.C., Ashok M.D., Yogesh T.J. (2005). *Bacopa monnieri* (L.) Pennell: A rapid, efficient and cost effective micropropagation. Plant Tiss. Cultbi. Otech..

[27] Tiwari V., Tiwari K.N., Singh B.D. (2006). Shoot bud regeneration from different explants of *Bacopa monniera* (L.) Wettst. by trimethoprim and bavistin. Plant Cell Rep..

[28] Sharath R., Krishna V., Sathyanarayana B.N., Prasad B.M., Harish B.G. (2007). High frequency regeneration through somatic embryogenesis in *Bacopa monnieri* (L.) Wettest, an important medicinal plant. Med. Aromat Plant Sci. Biotechnol..

[29] Banerjee M., Shrivastava S. (2008). An improved protocol for in vitro multiplication of *Bacopa monnieri* (L.). World J. Microbiol. Biotechnol..

[30] Debnath M. (2008). Responses of *Bacopa monnieri* to salinity and drought stress *in vitro*. J. Med. Plants Res..

[31] Praveen N., Naik P.M., Manohar S.H., Nayeem A., Murthy H.N. (2009). In vitro regeneration of brahmi shoots using semisolid and liquid cultures and quantitative analysis of bacoside A. Acta Physiol. Plant.

[32] Banerjee M., Modi P. (2010). Micropropagation of *Bacopa monnieri* using cyanobacterial liquid medium. Plant Tiss. Cult. Biotech..

[33] Ceasar S.A., Maxwell S.L., Prasad K.B., Karthigan M., Ignacimuthu S. (2010). Highly efficient shoot regeneration of *Bacopa monnieri* (L.) using a two-stage culture procedure and assessment of genetic integrity of micropropagated plants by RAPD. Acta Physiol. Plant.

[34] Joshi A.G., Pathak A.R., Sharma A.M., Singh S. (2010). High frequency of shoot regeneration on leaf explants of *Bacopa monnieri*. Environ. Exp. Biol..

[35] Parale A., Barmukh R., Nikam T. (2010). Influence of organic supplements on production of shoot and callus biomass and accumulation of bacoside in *Bacopa monniera* (L.) Pennell. Physiol. Mol. Biol. Plants.

[36] Sharma S., Kamal B., Rathi N., Chauhan S., Jadon V., Vats N., Gehlot A., Arya S. (2010). In vitro rapid and mass multiplication of highly valuable medicinal plant *Bacopa monnieri* (L.) Wettst. Afr. J. Biotechnol..

[37] Rout J.R., Sahoo S.L., Ray S.S., Sethi B.K., Das R. (2011). Standardization of an efficient protocol for in vitro clonal propagation of *Bacopa monnieri* L.—An important medicinal plant. J. Agric. Tech..

[38] Jain N., Sharma V., Ramawat K.G. (2012). Shoot culture of *Bacopa monnieri*: Standardization of explant, vessels and bioreactor for growth and antioxidant capacity. Physiol. Mol. Biol. Plants.

[39] Mehta J., Ansari R., Syedy M., Khan S., Sharma S., Gupta N., Rathore R., Vaishnav K. (2012). An effective method for high frequency multiple shoots regeneration and callus induction of *Bacopa monnieri* (L.) Pennel: An important medicinal plant. Asian J. Plant Sci. Res..

[40] Pandiyan P., Selvaraj T. (2012). In vitro multiplication of *Bacopa monnieri* (L.) Pennell from shoot tip and nodal explants. J. Agric. Tech..

[41] Rao S., Rajkumar P., Kaviraj C., Parveen P.A. (2012). Efficient plant regeneration from leaf explants of *Bacopa monniera* (L.) Wettst.: A threatened medicinal herb. Ann. Phytomed.

[42] Sharma M., Raina H., Verma V., Mallubhotla S., Ahuja A. (2012). Synthetic seeds a viable approach for conservation and propagation of phytoremediant herb: *Bacopa monnieri* (L.) Wettst. J. Env. Res. Dev..

[43] Tiwari K.N., Tiwari V., Singh J., Singh B.D., Ahuja P. (2012). Synergistic effect of trimethoprim and bavistin for micropropagation of *Bacopa monniera*. Biol. Plant.

[44] Bhusari S., Wanjari R., Khobragade P. (2013). Cost effective in vitro clonal propagation of *Bacopa monnieri* L.. Penell. Int. J. Indig. Med. Plants.

[45] Ghasolia B., Shandilya D., Maheshwari R. (2013). Multiple shoot regeneration of *Bacopa monnieri* (L.) using cyanobacterial media- a novel approach and effect of phytoregulators on in vitro micropropagation. Int. J. Rec. Biotechnol..

[46] Jain R., Prasad B., Jain M. (2013). In vitro regeneration of *Bacopa monnieri* (L.): A highly valuable medicinal plant. Int. J. Curr. Microbiol. Appl. Sci..

[47] Karatas. M., Aasim M., Dogan M., Khawar K.M. (2013). Adventitious shoot regeneration of the medicinal aquatic plant water hyssop (*Bacopa monnieri* L. Pennell) using different internodes. Arch. Biol. Sci..

[48] Kaur J., Nautiyal K., Pant M. (2013). In vitro propagation of *Bacopa monnieri* (L.) Wettst A medicinally priced herb. Int. J. Curr. Microbiol. Appl. Sci..

[49] Begum T., Mathur M. (2014). In vitro regeneration of *Catharanthus roseus* and *Bacopa monnieri* and their survey around Jaipur District. Int. J. Pure Appl. Biosci..

[50] Jain A., Pandey K., Benjamin D., Meena A.K., Singh R.K. (2014). In vitro approach of medicinal herb: *Bacopa monnieri*. Int. J. Innov. Res. Sci. Eng. Technol..

[51] Karatas M., Aasim M. (2014). Efficient in vitro regeneration of medicinal aquatic plant water hyssop (*Bacopa monnieri* L. Pennell). Pak. J. Agric. Sci..

[52] Naik P.M., Patil B.R., Kotagi K.S., Kazi A.M., Lokesh H., Kamplikoppa S.G. (2014). Rapid one step protocol for in vitro regeneration of *Bacopa monnieri* (L.). J. Cell Tissue Res..

[53] Umesh T.G., Sharma A., Rao N. (2014). Regeneration potential and major metabolite analysis in nootropic plant- *Bacopa monnieri* (L.) Pennell. Asian J. Pharm. Clin. Res..

[54] Behera S., Nayak N., Shasmita D.P., Naik S.K. (2015). An efficient micropropagation protocol of *Bacopa monnieri* (L.) Pennell through two-stage culture of nodal segments and *ex vitro* acclimatization**. J. Appl. Biol. Biotech..

[55] Mishra S.K., Tiwari K.N., Shivna P.L., Mishra A.K. (2015). Micropropagation and comparative phytochemical, antioxidant study of *Bacopa monnieri* (L.) Pennell. Res. J. Pharm. Biol. Chem. Sci..

[56] Sharma M., Gupta R., Khajuria R.K., Mallubhotla S., Ahuja A. (2015). Bacoside biosynthesis during in vitro shoot multiplication in *Bacopa monnieri* (L.) Wettst. grown in Growtek and air lift bioreactor. Indian J. Biotechnol..

[57] Croom L.A., Jackson C.L., Vaidya B.N., Parajuli P., Joshee N. (2016). Thin Cell Layer (TCL) Culture System for Herbal Biomass Production and Genetic Transformation of *Bacopa monnieri* L. Wettst. Am. J. Plant Sci..

[58] Hegazi G.A.E.M. (2016). In vitro preservation of *Bacopa monnieri* (L.) Pennell as a rare medicinal plant in Egypt. J. Basic Appl. Sci. Res..

[59] Karatas M., Aasim M., Dazkirli M. (2016). Influence of light-emitting diodes and benzylaminopurin on adventitious shoot regeneration of water hyssop (*Bacopa monnieri* (L.) Pennell) *in vitro*. Arch. Biol. Sci..

[60] Khilwani B., Kaur A., Ranjan R., Kumar A. (2016). Direct somatic embryogenesis and encapsulation of somatic embryos for in vitro conservation of *Bacopa monnieri* (L.) Wettst. Plant Cell Tiss. Organ Cult..

[61] Pothiaraj G., Ebenezer R.S., Christdas E.J., Shakila H. (2016). Comparative analysis on the effect of seaweed liquid extracts and commercial plant growth regulators on in vitro propagation of *Bacopa monnieri*. Int. J. Res. Biol. Sci..

[62] Wangdi K., Sarethy I.P. (2016). Evaluation of micropropagation system of *Bacopa monnieri* L. in liquid culture and its effect on antioxidant properties. J. Herbs Spices Med. Plants.

[63] Haque S.K.M., Chakraborty A., Dey D., Mukherjee S., Nayak S., Ghosh B. (2017). Improved micropropagation of *Bacopa monnieri* (L.) Wettst. (Plantaginaceae) and antimicrobial activity of in vitro and *ex vitro* raised plants against multidrug-resistant clinical isolates of urinary tract infecting (UTI) and respiratory tract infecting (RTI) bacteria. Clin. Phytosci..

[64] Rency A.S., Satish L., Pandian S., Rathinapriya P., Ramesh M. (2017). In vitro propagation and genetic fidelity analysis of alginate-encapsulated *Bacopa monnieri* shoot tips using Gracilariasalicornia extracts. J. Appl. Phycol..

[65] Sarkar S., Jha S. (2017). Morpho-histological characterization and direct shoot organogenesis in two types of explants from *Bacopa monnieri* on unsupplemented basal medium. Plant Cell Tiss. Organ Cult..

[66] Faisal M., Alatar A.A., El-Sheikh M.A., Abdel-Salam E.M., Qahtan A.A. (2018). Thidiazuron induced in vitro morphogenesis for sustainable supply of genetically true quality plantlets of Brahmi. Ind. Crop. Prod..

[67] Murashige T., Skoog F. (1962). A revised medium for rapid growth and bioassays with tobacco tissue cultures. Physiol. Plant.

[68] Goel A., Kaur A., Kumar A. (2018). Biochemical and histological changes during in vitro rooting of microcuttings of Bacopa monnieri (L.) Wettst. Acta Physiol. Plant.

[69] Ikeuchi M., Ogawa Y., Iwase A., Sugimoto K. (2016). Plant regeneration: Cellular origins and molecular mechanisms. Development.

[70] Deepa A.V., Anju M., Dennis Thomas T., Ahmad N., Faisal M. (2018). The Applications of TDZ in Medicinal Plant Tissue Culture. Thidiazuron: From Urea Derivative to Plant Growth Regulator.

[71] Dinani E.T., Shukla M.R., Turi C.E., Sullivan J.A., Saxena P.K., Ahmad N., Faisal M. (2018). Thidiazuron: Modulator of Morphogenesis *In vitro*. Thidiazuron: From Urea Derivative to Plant Growth Regulator.

[72] Subashri B., Pillai Y.J.K. (2014). In vitro regeneration of *Bacopa monnieri* (L.) Pennel.- A multipurpose medicinal plant. Int. J. Pharm. Sci..

[73] Jha S., Sen S. (1987). Nuclear changes and organogenesis during callus culture of *Urginea indica* Kunth., Indian squill. Cytologia.

[74] Jha S., Sen S. (1990). Induction of mitosis in polytene nuclei and hormonal effect on nuclear changes during callus initiation in diploid *Urginea indica* Kunth. (liliaceae). Genetica.

[75] Samaddar T., Nath S., Halder M., Sil B., Roychowdhury D., Sen S., Jha S. (2012). Karyotype analysis of three important traditional Indian medicinal plants, *Bacopa monnieri*, Tylophoraindica and Withaniasomnifera. Nucleus.

[76] Dey A., Hazra A.K., Nongdam P., Nandy S., Tikendra L., Mukherjee A., Banerjee S., Mukherjee S., Pandey D.K. (2019). Enhanced Bacoside content in polyamine treated in vitro raised Bacopa monnieri (L.) Wettst. S. Afr. J. Bot..

[77] Largia M.J., Pothiraj G., Shilpha J., Ramesh M. (2015). Methyl jasmonate and salicylic acid synergism enhances bacoside A content in shoot cultures of *Bacopa monnieri* (L.). Plant Cell Tiss. Organ Cult..

[78] Chakravarty A.K., Sarkar T., Nakane T., Kawahara N., Masuda K. (2002). New phenylethanoid glycosides from *Bacopa monniera*. Chem. Pharm. Bull..

[79] Hou C.C., Lin S.J., Cheng J.T., Hsu F.L. (2002). Bacopaside III, bacopasaponin G and bacopasides A, B, and C from *Bacopa monniera*. J. Nat. Prod..

[80] Deepak M. (2003). The need for establishing identities of ‘bacoside A and B’? The putative major bioactive saponins of Indian medicinal plant. Phytomedicine.

[81] Singh H.K., Rastogi R.P., Srimal R.C., Dhawan B.N. (1988). Effect of bacosides A and B on avoidance responses in rats. Phytother. Res..

[82] Singh H.K., Dhawan B.N. (1997). Neuropsychopharmacological effects of the Ayurvedic nootropic *Bacopa monniera* Linn. (Brahmi). Indian J. Pharm..

[83] Kulshreshtha D.K., Rastogi R.P. (1973). Bacogenin-A1: A novel dammarane triterpene sapogenin from *Bacopa monniera*. Phytochemistry.

[84] Singh H.K., Dhawan B.N. (1982). Effect of *Bacopa monnieri* Linn. (Brahmi) extract on avoidance responses in rat. J. Ethnopharmacol..

[85] Jain P., Kulshreshtha D.K. (1993). Bacoside A1, A minor saponin from *Bacopa monniera*. Phytochemistry.

[86] Rastogi S., Pal R., Kulshreshtha D.K. (1994). Bacoside A3-a triterpenoid saponin from *Bacopa monniera*. Phytochemistry.

[87] Chakravarty A.K., Garai S., Masuda K., Nakane T., Kawahara N. (2003). Bacopasides III-V: Three new triterpenoid glycosides from *Bacopa monniera*. Chem. Pharm. Bull..

[88] Zhou Y., Kong D.Y., Peng L., Zhang W.D. (2009). A new triterpenoid saponin from *Bacopa monniera*. Chin. Chem. Lett..

[89] Murthy P.B., Raju V.R., Ramakrisana T., Chakravarthy M.S., Kumar K.V., Kannababu S., Subbaraju G.V. (2006). Estimation of twelve bacopa saponins in *Bacopa monnieri* extracts and formulations by high-performance liquid chromatography. Chem. Pharm. Bull.

[90] Garai S., Mahato S.B., Ohtani K., Yamasaki K. (1996). Dammarane type triterpenoid saponins from *Bacopa monniera*. Phytochemistry.

[91] Garai S., Mahato S.B., Ohtani K., Yamasaki K. (1996). Bacopasaponin D—A pseudojujubogenin glycoside from *Bacopa monniera*. Phytochemistry.

[92] Mahato S.B., Garai S., Chakravarty A.K. (2000). Bacopasaponins E and F: Two jujubogeninbisdesmosides from *Bacopa monniera*. Phytochemistry.

[93] Rastogi S., Kulshreshtha D.K. (1999). Bacoside A2–A triterpenoid saponin from *Bacopa monniera*. Ind. J. Chem..

[94] Chakravarty A.K., Sarkar T., Masuda K., Shiojima K., Nakane T., Kawahara N. (2001). Bacopaside I and II: Two pseudojujubogenin glycosides from *Bacopa monniera*. Phytochemistry.

[95] Mandal S., Mukhopadhyay S., Bacopasaponin H. (2004). A pseudojujubogenin glycoside from *Bacopa monniera*. Indian J. Chem..

[96] Bhandari P., Kumar N., Singh B., Kaur I. (2009). Dammarane triterpenoid saponins from *Bacopa monnieri*. Can. J. Chem..

[97] Peng L., Zhou Y., de Kong Y., Zhang W.D. (2010). Antitumor activities of dam- marane triterpene saponins from *Bacopa monniera*. Phytother. Res..

[98] Rohini G., Devi S. (2008). *Bacopa monniera* extract induces apoptosis in murine sarcoma cells (s-180). Phytother. Res..

[99] Charles P.D., Ambigapathy G., Geraldine P., Akbarasha M.A., Rajan K.E. (2011). *Bacopa monniera* leaf extract up-regulates tryptophan hydroxylase (TPH2) and serotonin transporter (SERT) expression: Implications in memory formation. J. Ethnopharmacol..

[100] Ahirwar S., Tembhre M., Gour S., Namdeo A. (2012). Anticholinesterase efficacy of *Bacopa monnieri* against the brain regions of rat—a novel approach to therapy for Alzheimer’s disease. Asian J. Exp. Sci..

[101] Raghav S., Singh H., Dalal P.K., Srivastava J.S., Asthana O.P. (2006). Randomized controlled trial of standardized *Bacopa monniera* extract in age- associated memory impairment. Indian J. Psychiatry.

[102] Viji V., Helen A. (2008). Inhibition of lipoxygenases and cyclooxygenase-2 en- zymes by extracts isolated from *Bacopa monniera* (L.) Wettst. J. Ethnopharmacol..

[103] Anbarasi K., Kathirvel G., Vani G., Jayaraman G., Devi S.C.S. (2006). Cigarette smoking induces heat shock protein 70 kDa expression and apoptosis in rat brain: Modulation by bacoside A. Neuroscience.

[104] Janani P., Sivakumari K., Parthasarathy C. (2009). Hepatoprotective activity of bacoside A against N-nitrosodiethylamine-induced liver toxicity in adult rats. Cell Biol. Toxicol..

[105] Anbarasi K., Sabitha K.E., Devi C.S.S. (2005). Lactate dehydrogenase isoenzyme patterns upon chronic exposure to cigarette smoke: Protective effect of bacoside A. Environ. Toxicol. Pharmacol..

[106] Anbarasi K., Vani G., Balakrishna K., Devi S.C.S. (2005). Creatine kinase isoenzyme patterns upon chronic exposure to cigarette smoke: Protective effect of bacoside A. Vasc. Pharm..

[107] Rauf K., Subhan F., Abbas M., Badshah A., Ullah I., Ullah S. (2011). Effect of bacopasides on acquisition and expression of morphine tolerance. Phytomedicine.

[108] Uabundit N., Wattanathorn J., Mucimapura S., Ingkaninan K. (2010). Cognitive enhancement and neuroprotective effects of *Bacopa monnieri* in Alzheimer’s disease model. Journal of Ethnopharmacology..

[109] Kikusaki H., Nakatani N. (1993). Antioxidant effect of some ginger constituents. J. Food Sci..

[110] Vohra S.B., Khanna T., Athar M., Ahmed B. (1997). Analgesic activity of bacosine, a new triterpene isolated from *Bacopa monnieri*. Fitoterapia.

[111] Tripathi Y.B., Chaurasia S., Tripathi E., Upadhyay A., Dubey G.P. (1996). *Bacopa monniera* Linn. as an antioxidant: Mechanism of action. Indian J. Exp. Biol..

[112] Bhattacharya S.K., Bhattacharya A., Kumar A., Ghosal S. (2000). Antioxidant activity of *Bacopa monniera* in rat frontal cortex, striatum and hippocampus. Phytother. Res..

[113] Pawar R., Gopalakrishnan C., Bhutani K.K. (2001). Dammarane triterpene saponin from *Bacopa monniera* as the superoxide inhibitor in polymorphonuclear cells. Planta Med..

[114] Sumathy T., Subramanian S., Govindasamy S., Balakrishna K., Veluchamy G. (2001). Protective role of *Bacopa monniera* on morphine induced hepatotoxicity in rats. Phytother. Res..

[115] Volluri S.S., Bammidi S.R., Chippada S.C., Vangalapati M. (2011). In vitro antioxidant activity and estimation of total phenolic content in methanolic extract of *Bacopa monniera*. Rasayan J. Chem..

[116] Mallick M.N., Akhtar M.S., Najm M.Z., Tamboli E.T., Ahmad S., Husain S.A. (2015). Evaluation of anticancer potential of *Bacopa monnieri* L. against MCF-7 and MDA-MB 231 cell line. J. Pharm. Bioallied. Sci..

[117] Pawar R.S., Khan S.I., Khan I.A. (2007). Glycosides of 20-deoxy derivatives of jujubogenin and pseudojujubogenin from *Bacopa monniera*. Planta Med..

[118] Sivaramakrishna C., Rao C.V., Trimurtulu G., Vanisree M., Subbaraju G.V. (2005). Triterpenoid glycosides from *Bacopa monnieri*. Phytochemistry.

[119] Mclaughlin J.L., Rogers L.L., Anderson J.E. (1998). The uses of biological assays to evaluate botanicals. Drug Inf. J..

[120] D’Souza P., Deepak M., Rani P., Kadamboor S., Mathew A., Chandrashekar A.P., Agarwal A. (2002). Brine shrimp lethality assay of *Bacopa monnieri*. Phytother. Res..

[121] Jain P., Khanna N.K., Trehan N., Pendse V.K., Godhwani J.L. (1994). Antiinflammatory effects of an Ayurvedic preparation, Brahmi Rasayan, in rodents. Indian J. Exp. Biol..

[122] Holcomb L.A., Dhanasekaran M., Hitt A.R., Young K.A., Riggs M., Manyam B.V. (2006). *Bacopa monniera* extract reduces amyloid levels in PSAPP mice. J. Alzheimers Dis..

[123] Ajalus S.M., Chakma N., Rahman M., Salahuddin M., Kumar S.S. (2013). Assessment of analgesic, antidiarrhoeal and cytotoxic activity of ethanolic extract of the whole plant of *Bacopa monnieri* Linn. Int. Res. J. Pharm..

[124] Mathur A., Verma S.K., Purohit R., Singh S.K., Mathur D., Prasad G., Dua V.K. (2010). Pharmacological investigation of *Bacopa monnieri* on the basis of antioxidant, antimicrobial and anti-inflammatory properties. J. Chem. Pharm. Res..

[125] Rao C.V., Sairam K., Goel R.K. (2000). Experimental evaluation of Bocopamonniera on rat gastric ulceration and secretion. Indian J. Physiolpharmacol..

[126] Subhan F., Abbas M., Rauf K., Arfan M., Sewell R.D., Ali G. (2010). The role of opioidergic mechanism in the activity of *Bacopa monnieri* extract against tonic and acute phasic pain modalities. Pharmacologyonline.

[127] Devishree R.A., Saravana K., Ashish R. (2017). Short term effect of *Bacopa monnieri* on memory—A brief review. J. Pharm. Res..

[128] Knopman D.S. (2006). Current treatment of mild cognitive impairment and Alzheimer’s disease. Curr. Neurol. Neurosci. Rep..

[129] Goswami S., Saoji A., Kumar N., Thawani V., Tiwari M., Thawani M. (2011). Effect of Bacopa monnieri on cognitive functions in Alzheimer’s disease patients. Int. J. Collab. Res. Intmed. Public Health.

[130] Samanta D., Mallick B., Roy D. (2019). In vitro clonal propagation, organogenesis and somaclonal embryogenesis in *Bacopa monnieri* (L.) Wettst. Plant Sci..

